# Course and Prognosis of AA Amyloidosis in Patients with Psoriatic Arthritis: Report of Three Cases from a Single Center Cohort and Review of the Literature

**DOI:** 10.31138/mjr.33.2.185

**Published:** 2022-06-30

**Authors:** Murat Bektaş, Nevzat Koca, Burak Ince, Yasemin Yalçınkaya, Bahar Artım Esen, M. Lale Öcal, Ahmet Gül, Murat Inanç

**Affiliations:** Division of Rheumatology, Department of Internal Medicine Istanbul Faculty of Medicine, Istanbul University, Çapa, Istanbul, Turkey

**Keywords:** psoriatic arthritis, AA amyloidosis, MEFV variants

## Abstract

**Objective:**

Herein, we aimed to evaluate the frequency and clinical features of AA amyloidosis in patients with PsA followed up in our tertiary referral clinic.

**Methods:**

We retrospectively evaluated PsA patients classified according to CASPAR classification criteria followed-up in our tertiary referral clinic for AA amyloidosis. The literature search was also done by three independent researchers using the keywords “psoriatic arthritis AND amyloidosis”, “spondyloarthritis AND amyloidosis”, “AA amyloidosis”, “secondary amyloidosis”.

**Results and conclusions:**

A total of 253 patients were included into the analysis. Two thirds of (n=162; 64%) the patients were women, and the mean age of the patients was 50.6 ± 13.4 (range, 20–90). We identified three patients with AA amyloidosis in 253 patients with PsA (1.2 %). The frequency of PsA-related amyloidosis in our AA amyloidosis cohort (n=165) was 1.8 %. Literature search revealed only a retrospective cohort study and 17 case reports, and we analysed these 31 cases. Nearly half of the cases were male, mean age of the patients was 50.7±15.3 and mean age of amyloidosis diagnosis was 47.2±16.7 years. Most of these patients had both polyarticular and axial involvement (81.3%). AA amyloidosis is a rare in patients with PsA. It should be kept in mind that patients with PsA who have not received appropriate treatment for a long time and/or have refractory disease may develop AA amyloidosis.

## BACKGROUND

Psoriatic arthritis (PsA) is a chronic inflammatory disease associated with psoriasis. Its prevalence is 1–2/1000 and is developed in 4–30% of patients with psoriasis. PsA is characterised by five different joint involvement patterns such as distal interphalangeal arthritis, asymmetric oligoarthritis, spondylitis, polyarthritis and arthritis mutilans accompanying dactylitis, tenosynovitis, enthesitis, skin and nail involvement.^1^ PsA patients have increased risk for permanent deformities, workforce loss, decreased quality of life and increased mortality.^2^ Thus, early diagnosis and treatment of PsA is important for the improvement of prognosis and prevention of joint damage.

Amyloid-A (AA) amyloidosis is a rare disorder developing as a consequence of uncontrolled chronic inflammatory diseases.^3^ Extracellular deposition of the cleaved products of soluble acute-phase reactant serum amyloid A (SAA) protein monomers as insoluble polymeric amyloid fibrils results in organ damage.^4^ AA amyloidosis has been associated with increased serum concentrations of SAA in uncontrolled chronic inflammatory diseases such as rheumatoid arthritis (RA), ankylosing spondylitis (AS), inflammatory bowel disease and hereditary periodic fever syndromes (HPFS).^4^

The availability of new treatments over the last decade, including biologic disease modifying antirheumatic drugs (b-DMARD) such as inhibitors of TNF-α (anti-TNFs), have revolutionized the treatment, demonstrating improved efficacy in controlling the disease activity of patients with inflammatory rheumatic diseases and resistant or intolerant to conventional synthetic DMARDs.^5^ Controlling the underlying disease activity and suppression of the acute phase response are essential in the treatment of AA amyloidosis accompanying a chronic inflammatory disease. While colchicine and IL-1 antagonists are effective in AA amyloidosis associated with HPFS^6–7^; TNF inhibitors, IL-6 antagonists and other b-DMARDs are used in addition to cs-DMARDs in AA amyloidosis associated with RA and other inflammatory diseases for this purpose.^8–9^

PsA is a relatively common inflammatory arthropathy, but there is limited data about its association with AA amyloidosis consisting only of small observational studies and case reports^10–11^; therefore, the frequency of AA amyloidosis in PsA is not clear.^12^ In this study, we aimed to evaluate the frequency and clinical features of AA amyloidosis in patients with PsA followed up in our tertiary referral clinic.

## PATIENTS AND METHODS

We retrospectively evaluated PsA patients classified according to CASPAR classification criteria followed-up in our tertiary referral clinic for the features of AA amyloidosis. Clinical data were collected with standardised forms from the charts of patients with psoriatic arthritis and amyloidosis. When necessary, subcutaneous, rectal or kidney biopsy was performed and diagnosis of AA amyloidosis was made by birefringence by Congo Red staining, and AA type amyloidosis was confirmed by immunohistochemistry. Patients with a clinical diagnosis of FMF and/or other inflammatory diseases were excluded from the study.

The literature search was done by three independent researchers using the keywords “psoriatic arthritis AND amyloidosis”, “spondyloarthritis AND amyloidosis”, “AA amyloidosis”, “secondary amyloidosis”.

For statistical analysis, SPSS (Statistical Package for the Social Sciences) program (v21.0, IBM, Armonk, NY, USA) was used. Descriptive statistics, discrete and continuous numerical variables were expressed as mean, ± standard deviation or median (minimum–maximum). Categorical variables were expressed as number of cases and percentages.

Local Ethics Committee reviewed and approved the study protocol (2019/1359).

## RESULTS

A total of 253 patients with the diagnosis of PsA were identified from the retrospective chart screen and included into the analysis. Nearly two thirds of (n=162; 64%) the patients were women, and the mean age of the patients was 50.6 ± 13.4 (range, 20–90) years. Mean follow-up of patients was 11.9 ± 7.9 years, the mean duration of psoriasis (PsO) was 20.5 ± 11.9 (range, 0.5–62) years and mean age of onset of PsA was 38.2 ± 12.1 (range, 11–77) years. The most common form of PsA was the oligo/monoarthritis type (40.2%) in the cohort. While most of the patients were on methotrexate (77.9%), seventy patients (27.7%) received TNF inhibitors for active PsA. Clinical and laboratory characteristics of patients with PsA were summarised in **Table 1**.

**Table 1.** Baseline clinical characteristics of 253 patients with psoriatic arthritis cohort.

**Clinical Variables**	**n (%)**	**Mean ± SD**	**Range**

**Age (year)**		50.58±13.46	20–90

**Sex**			
Female	162 (64)		
Male	91 (36)		

**Age of PsA onset (years)**		38.3±12.1	11–77

**Duration of PsA (years)**		12.0±7,9	0.5–43

**Duration of PsO (years)**		20.6±11.9	0.5–62

**Number of joints involved**		3.6±2.0	1–14

**CRP (mg/L)**		10.9±1.4	0–309

**ESR (mm/hour)**		26.7±24.0	0–120

**Methotrexate use (%) (oral + parenteral)**	197 (77.9)		

**Leflunomide use**	25 (9.9)		

**Sulfasalazine use**	59 (23.3)		

**Hydroxy-chloroquine use**	8 (3.2)		

**Received anti-TNF treatment**	70 (27.7)		

**Others**	1 (0.4)		

Others: cyclosporine or azathioprine; PsA: Psoriatic arthritis; PsO: Psoriasis; CRP: C reactive protein; ESR: Erythrocyte sedimentation rate; SD: Standard deviation.

We identified three patients with AA amyloidosis in 253 patients with PsA (1.2 %). The prevalence of PsA-related amyloidosis in our AA amyloidosis cohort (n=165) was 1.8% (**Table 2**). Clinical and laboratory characteristics of the patients are described in **Table 3**. Axial involvement, coexisting heterozygous *MEFV* gene mutation, polyarticular subtype of PsA were observed in two patients with PsA-related AA amyloidosis. Patient with the *MEFV* gene p.Met694Val variant was the youngest one among the three cases with amyloidosis.

**Table 2.** Baseline demographic and clinical characteristics of 165 patients with AA amyloidosis associated with several inflammatory diseases.

**Clinical and laboratory variables**	**n (%)**	**Mean ± SD**	**Range**

**Age**	165	45.4±12.7	21–78

**Sex**			
Male	85 (51.5)		
Female	80 (48.5)		

**Age of onset (FMF)**		25.9±14.2	

**Family history of FMF**	92 (59)		

**Duration of Amyloidosis (months)**		130.9±99	1–504

**Age of onset (Amyloidosis)**		33.5±14	5–70

**Etiology of Amyloidosis**			
FMF	128 (77.6)		
AS	8 (4.8)		
Idiopathic	13 (7.9)		
RA	2 (1.2)		
TRAPS	2 (1.2)		
Takayasu	2 (1.2)		
DADA2	2 (1.2)		
GPA	1 (0.6)		
Crohn’s disease	1 (0.6)		
AOSD	1 (0.6)		
PsA	3 (1.8)		
Gout	1 (0.6)		
Behçet’s disease	1 (0.6)		

FMF: Familial Mediterranean fever; AS: Ankylosing spondylitis; RA: Rheumatoid arthritis; TRAPS: TNF receptor associated periodic syndrome; DADA2: Deficiency of adenosine deaminase 2; GPA: Granulomatosis with polyangiitis; AOSD: Adult onset still disease; PsA: Psoriatic arthritis; SD: Standard deviation.

**Table 3.** Clinical and laboratory characteristics of three patients with PsA-related AA amyloidosis.

**Clinical and laboratory variables**	**Patient 1**	**Patient 2**	**Patient 3**
**Age (years)**	55	47	61
**Age of PsA onset (years)**	18	21	47
**Age of amyloidosis diagnosis (years)**	27	32	57
**Subtype of PsA**	Polyarthritis	Polyarthritis	Oligoarthritis
**Duration (years) and subtypes of psoriasis**	37 (plaque)	35 (plaque)	4 (plaque)
**Axial involvement**	Present	No	Present
**MEFV mutation**	M694V heterozygous	E148Q heterozygous	Negative
**Family history**	AS + Amyloidosis (sibling)	No	No
**Confirmed organ involvement of amyloidosis**	Kidney Gastrointestinal	Kidney	Kidney Gastrointestinal
**CRP (mg/L) (baseline)**	48	38	80
**ESR (mm/hour) (baseline)**	90	87	95
**Creatinine (mg/dL) (baseline)**	0.7*	0.9*	0.5
**Proteinuria (g/day) (baseline)**	2.1	8	4.2
**History of cs-DMARDs and other immunosuppressives**	Cyclosporine Cyclophosphamide Azathioprine Sulfasalazine Leflunomide Colchicine	Methotrexate Colchicine	Methotrexate
**History of b-DMARD treatment**	Etanercept Certolizumab	None	Etanercept Adalimumab Certolizumab Infliximab Secukinumab
**Current treatment**	Tacrolimus Mycophenolate mofetil Prednisolone 5 mg/day	Tacrolimus Mycophenolate mofetil Prednisolone 5 mg/day	Secukinumab 300 mg/month
**Amyloidosis outcome**	ESRD Renal tx	ESRD Renal tx	Partial response

* After renal transplantation; PsA: Psoriatic arthritis; cs-DMARD: Conventional synthetic disease modifying antirheumatic drug; b-DMARD: Biological disease modifying antirheumatic drug; CRP: C-reactive protein; ESR: Erythrocyte sedimentation rate; ESRD: End stage renal disease; renal tx: Renal transplantation.

All three patients received at least one cs-DMARD and two patients (patient 1 and patient 3) have treated with multiple b-DMARDs during the clinical course. In addition, these three patients had a period over 10 years (mean 13.3 years) without a specific DMARD treatment during their clinical course before attendance to our clinic due to late referral or lost to follow-up. Development of end stage renal disease and need for renal transplantation were observed in two patients (patient 1 and patient 2). Two patients were followed-up without proteinuria after renal transplantation, although patient 1 remained to have increased CRP levels. Patient 3 was unresponsive to multiple b-DMARDs and had a partial response to high-dose (300 mg/month) secukinumab treatment at the last visit.

## CASE REPORT 1

A 55-year-old man with a history of pulmonary tuberculosis applied to our clinic with symmetrical polyarthritis, inflammatory back pain and psoriatic skin lesions simultaneously in 1983. Rheumatoid factor (RF) was negative, and x-ray showed unilateral sacroiliitis. After failure of cyclosporine, he also became resistant to a short course of cyclophosphamide and then azathioprine.

Between 1983–1993 he had high disease activity with elevated acute phase reactants. In 1993, he developed 2.1 g/day proteinuria; and kidney biopsy revealed AA amyloidosis. The *MEFV* gene analysis showed heterozygous p.Met694Val variant on exon 10 without any symptom or family history for FMF. Sulfasalazine, leflunomide and colchicine treatments were ineffective after diagnosis of amyloidosis. He developed end stage renal disease and underwent renal transplantation five years ago. During the follow-up, he developed chronic diarrhoea, and histopathological examination of the colonoscopic biopsy from rectum confirmed AA amyloidosis. He has been followed up with tacrolimus 2 mg/day, prednisolone 5 mg/day, mycophenolate mofetil 2000 mg/day and colchicine 1 mg/day with mild disease activity, normal creatinine levels and without proteinuria.

## CASE REPORT 2

Forty-seven-year-old man presented with polyarthritis and history of psoriasis (nine years) when he was 21, and he was negative for RF. He started to receive methotrexate (MTX) and low dose glucocorticoids (GCs) with the diagnosis of PsA, but he had poor adherence to follow-up and medications. The patient was admitted to our clinic because of peripheral oedema when he was 32 years old. Nephrotic range proteinuria (8 g/day) was detected, and kidney biopsy confirmed AA amyloidosis. The *MEFV* gene analysis revealed heterozygous p.Glu148Gln variant on exon 2 without any symptom or family history of FMF. Colchicine 1.5 mg/day was added to his treatment, he frequently missed the follow-up appointments. He underwent renal transplantation due to end stage renal disease when he was 45 years old. The patient has been treated with tacrolimus 1.5 mg/day, prednisolone 5 mg/day, mycophenolate mofetil 1500 mg/day, colchicine 1 mg/day; and he has no active disease, normal creatinine and CRP levels and no proteinuria for two years.

## CASE REPORT 3

A 61-year-old man applied to another rheumatology clinic because of oligoarthritis and axial involvement fourteen years ago, and he was treated with MTX 15 mg/week. He had poor adherence to his treatment at the beginning, but he later developed a refractory disease despite regular use of MTX. During his evaluation in 2016, he had oligoarthritis, inflammatory back pain and psoriatic skin lesions. RF and anti-CCP tests were negative, and he had bilateral sacroiliitis with hip involvement on pelvic X-ray. Adalimumab (ADA) treatment was ineffective; and switching to first etanercept (ETA) and thereafter to certolizumab (CZP) was also showed no clinical efficacy. Nephrotic range proteinuria continued when he was on CZP treatment, and histopathologic examination of colonoscopic biopsy from rectum revealed AA amyloidosis three years ago. CZP was then switched to infliximab (IFX). IFX was also ineffective, and he later started to receive secukinumab 150 mg monthly after the loading doses, 15 months ago. Since he had a partial response, the secukinumab dose was increased to 300 mg monthly, and clinical findings of both axial and peripheral arthritis had resolved, and an improvement in acute phase response and proteinuria was observed at the last visit.

## LITERATURE REVIEW

After the titles of the articles were scanned, the abstracts and full papers of the relevant articles were reviewed. Articles in languages other than English and whose abstracts could not be reached (n=18) were excluded from the study. Therefore, one retrospective cohort study and (13) and 17 case reports consisted of 31 patients were included to the study. The features of these cases were summarised in **Table 4**. Nearly half of the patients (45.8%) were male (11/24; not available in 7 cases). The mean age of the patients was 50.7±15.3 (range, 25–75), mean diagnosis age of PsA was 34.8±14 (range, 8–63), mean diagnosis age of amyloidosis was 47.2±16.7 (range, 19–71), and mean duration of PsA was 18.4±12.1 (range, 4–49) years. Among these patients both polyarticular and axial involvement were observed in 13 (81.3%) of 16 patients whose data were available (two patients had oligoarthritis, and one patient had arthritis mutilans). Kidney involvement was observed in 21 patients (data available in 24 patients) and one third of them (n=7) developed end stage renal disease. Gastrointestinal involvement was established in 10 patients. Other less frequent organ involvements such as spleen, heart, thyroid, and lungs were generally asymptomatic and detected at autopsy. Mortality was observed in 47.8% (11 of 23 patients) of the reported patients.

**Table 4.** Review of the patients with PsA-associated AA amyloidosis in the literature.

**Study name**	**Country**	**Year**	**Number of patients**	**Age**	**Sex**	**Age at diagnosis of PsA**	**Peripheral involvement of PsA**	**Axial involvement (yes/no)**	**Age at diagnosis of amyloidosis**	**Duration of PsA**	**Treatments before amyloidosis diagnosis**	**Location of amyloid deposits**	**Treatments received after amyloidosis diagnosis**	**Response to treatment**	**ESRD**	**Mortality**
Samantha Rodríguez-Muguruza et al.^22^	Spain	2015	Case number 1	71	M	22	Polyarticular	Yes	71	49	NA	Gallbladder, Kidney	IFX	No	Yes	Yes
			Case number 2	68	M	40	Oligoarticular	No	68	28	NA	Bladder	No	No	Yes	Yes
			Case number 3	33	F	29	Polyarticular	No	33	4	NA	Kidney	INF	Yes	No	No
			Case number 4	56	F	59	Yes	No	56	20	NA	Kidney, sc	No	Yes	No	No
K Immonen et al. study^21^	Finland	2011	Case number 1	36	F	8	Polyarticular	NA	19	28	MTX, GCs	sc*, colon*, kidney	MTX, GCs, ETA, Tocilizumab	Stable	No	No
			Case number 2	36	F	30	NA	NA	21	6	MTX, GCs	sc*, colon*, kidney	MTX, GCs, ETA, ADA	Stable	No	No
JC Nossent et al.^13^	Norway	2009	5 (329)	NA	NA	NA	Yes (all)	Yes (all)	NA	NA	NA	NA	NA	NA	NA	NA
Antonio Fernández-Nebro^46^	Spain	2005	Case number 1	60	M	19	NA	NA	55	41	NA	Kidney*	IFX	Stable	No	No
			Case number 2	48	F	28	NA	NA	45	20	NA	Kidney* , GIS, bladder	IFX, chlorambucil	No	No	Yes
			Case number 3	70	F	63	NA	NA	67	7	NA	Kidney* , peripheral nervous system	INF	Yes	No	No
			Case number 4	42	F	19	NA	NA	41	23	NA	Kidney*	ETA, GCs	Yes	No	No
M. A. Gertz et al.^47^	USA		Case number 1	75	F	NA	NA	NA	NA	NA	NA	Kidney*	MTX	Yes	No	No
			Case number 2	NA	NA	NA	NA	NA	NA	NA	NA	NA	NA	No	Yes	Yes
			Case number 3	NA	NA	NA	NA	NA	NA	NA	NA	NA	NA	No	Yes	Yes
Jacques-Eric Gottenberg et al.^11^	France	2003	1	68	M	51	Polyarticular	NA	67	17	None	Kidney*	IFX, AZA	Yes	No	No
Alexander Kagan et al.^17^	Israel	1999	1	25	F	15	Arthritis mutilans	NA	25	10	MTX, GCs	Kidney*	Colchicine	Yes	No	No
Anne Ferguson et al.^30^	Scotland	1968	1	63	F	43	Polyarticular	NA	63	20	NA	Small intestines*	NA	NA	NA	NA
Peter A. Berger et. al.^35^	Scotland	1969	1	31	M	23	Polyarticular	NA	29	8	MTX, GCs	Kidney, small intestine, adrenal glands, liver (necropsy)	MTX, GCs	No	Yes	Yes
M. David et al.^36^	Israel	1982	1	54	F	40	Polyarticular	Yes	54	14	AZA, GCs	Colon* , kidney, spleen, heart, stomach and intestine (necropsy)	NA	No	NA	Yes
S. Mpofu et al.^48^	United Kingdom	2003	1	53	M	32	Polyarticular	Yes	NA	21	SSZ, GCs,AZA, MTX, Cys,	Kidney* , spleen, liver	Chlorambucil	Yes	No	No
Dinoia et al.^16^	Italy	2016	1	50	M	29	NA	NA	50	21	SSZ, MTX, GCs,NSAIDs, IFX, ETA	Kidney*	Tocilizumab	No	No	Yes
Ujfalussy et al.^37^	Hungary	2001	1	63	F	54	Polyarticular	Yes	60	6	t	Buccal mucosa* , kidneys, pancreas, gastrointestinal tract, thyroid gland, heart, liver, lungs, and synovial membrane, skin (necropsy)	NA	No	No	Yes
JG Ryan et al.^14^	Ireland	2006	1	28	M	21	Oligoarticular	Yes	28	7	Gold, MTX, GCs, SSZ, Cys, AZA,	Kidney*	High dose Cys, GCs	No	Yes	Yes
Qureshi et al.^49^	United Kingdom	1977	1	50	M	36	Polyarticular	Yes	50	14	GCs	Small intestine* , rectum* , skin* , kidney (probably)	NA	NA	NA	NA
S.Tsuda et al.^50^	Japan	1995	1	38	M	35	Polyarticular	Yes	38	3	Etretinate, Cys,	Colon* , spleen, kidneys, liver, gastrointestinal tract, lung, heart, pancreas, adrenal glands, thyroid and gall-bladder(necropsy)	GCs, Dimethylsulfoxide	No	Yes	Yes
C. P. Willoughby et al.^31^	England	1981	1	35	M	31	Polyarticular	Yes	35	4	GCs, MTX, AZA, Dapson, Hydroxyurea, Methoxsalen,	Stomach,* intestine* , rectum*	Colchicine	Yes	NA	NA
C Fiehn et al. Study^24^	Germany	2003	1	64	F	34	Polyarticular	NA	64	30	NA	Kidney*	Infliximab	Yes	No	No

* diagnosis of amyloidosis verified with biopsy; NA: not available; M: male; F: female; MTX: methotrexate;

AZA: azathioprine; GCs: glucocorticoids; SSZ: sulfasalazine; Cys: cyclosporine; ETA: etanercept; ADA: adalimumab;

IFX: infliximab; NSAIDs: non-steroidal anti-inflammatory drugs; sc: subcutaneous tissue.

## DISCUSSION

AA amyloidosis is frequently associated with chronic infections such as tuberculosis and chronic inflammatory rheumatic conditions like RA and AS, previously. However widespread use of antibiotics and effective anti-rheumatic treatments have changed the course of the inflammatory diseases within the last three decades.^14–15^ Similarly, FMF is the most common disease associated with AA amyloidosis in Turkey due to its high prevalence in the Eastern Mediterranean, but its relative frequency decreased after effective use of colchicine treatment.^16–17^ There are few case reports showing the association between AA amyloidosis and psoriasis.^18–20^ Although the first case report of PsA-related AA amyloidosis was published in 1965, the frequency of AA amyloidosis associated with PsA and the characteristics of its clinical course have remained unclear.^21^ The frequency of amyloidosis in patients with PsA (1.2%) was found to be lower compared to the that of patients with FMF (9.9%) in our previous study.^22^ While FMF was the most common aetiology, the frequency of PsA was 3.95% (3 out of 76 patients) in patients with AA amyloidosis in a previous report from our centre.^23^ Immonen et al. reported three patients with AA amyloidosis in a study of 70 patients with PsA.^24^ In a retrospective analysis of 1125 patients with spondylarthritis from Spain, the frequency of amyloidosis was 1.3%. Although most patients with amyloidosis had AS, four of them had PsA (0.35 %).^25^

The treatment adherence problems in all three patients in our study supported the common opinion that long-term uncontrolled inflammation plays an important role in the development of AA amyloidosis. Furthermore, coexisting factors such as heterozygous mutant *MEFV* gene carriage and history of tuberculosis may have contributed to the development of AA amyloidosis. In an observational study from Turkey, heterozygosity for the *MEFV* gene p.Met694Val mutation had higher disease severity and longer attack duration compared to p.Glu148Gln mutation.^26^ Furthermore, p.Met694V variation was found to be more frequent in patients with seronegative spondylarthritis.^27–28^ In addition, higher frequency of p.Met694Val mutation heterozygosity was found in patients with AS, who developed amyloidosis compared to the frequency of p.Glu148Gln in our former study.^29^ Relatively earlier development of amyloidosis in patient 1 may be due to the presence of penetrating p.Met694Val *MEFV*. However, larger studies with higher numbers of patients are needed to further comment on the contribution of the *MEFV* gene variants and other factors to the amyloidosis risk.

Although there is limited data about the amyloidosis risk in association with PsA subtypes, the presence of axial involvement and polyarthritis in two of the three patients with amyloidosis in this series suggest that these two subtypes may have a higher risk for the development of amyloidosis due to the higher systemic inflammatory response. The fact that most of the PsA-related amyloidosis in the literature were reported in the axial and/or polyarticular subtype of PsA supports this finding.^30–32^ Two of our patients who were suffering from diarrhoea had biopsy-proven rectal amyloidosis. Gastrointestinal involvement of PsA-related amyloidosis was found in only two case reports in the literature.^33–34^ The frequency of gastrointestinal involvement in amyloidosis is unclear, and there is high possibility of underreporting. Involvement of many organs such as spleen, heart, gastrointestinal tract and bladder without specific symptoms was reported at autopsy investigations in many studies,^35–37^ which indicates that AA amyloidosis frequently affects many organs beyond the kidney. Therefore, screening for systemic involvement of AA amyloidosis may be important especially in symptomatic patient.

Although there is no consensus in the treatment of PsA-related amyloidosis, few case reports have been reported with positive experience by using biological drugs.^12,38^ In a case report of a patient with PsA-related amyloidosis, rapid improvement was observed with infliximab.^30^ In the study by Immonen et al., a favourable response was observed with tocilizumab and adalimumab in two patients.^24^ IL-17 plays a crucial role in the pathogenesis of spondylarthritis by causing enthesitis and bone resorption.^39^ Secukinumab is an anti-interleukin-17A monoclonal antibody that has been shown to control the symptoms of both AS and PsA.^40–41^ Secukinumab is effective for multiple domains of PsA, and it has been suggested as the first line b-DMARD similar to TNF inhibitors in the 2019 EULAR recommendations.^42^ Therefore, it can be speculated that targeting the underlying pathogenesis of PsA by IL-17 blockade may also contribute to the regression of amyloidosis. To our knowledge this study includes the first successful treatment experience with secukinumab in a refractory PsA-related amyloidosis. In patient 3, who was unresponsive to multiple anti-TNF drugs, satisfactory results both in controlling disease activity of PsA and amyloidosis were achieved with high dose secukinumab treatment.

The data about the outcome of amyloidosis patients with renal transplantation is also limited. In a multicentre retrospective study from France, the patient survival was found to be lower in kidney transplant recipients due to secondary amyloidosis compared to other reasons related kidney transplantation, although there was no difference in the graft survival.^43^ In an observational study from Turkey both graft and patient survival were lower in patients with FMF-related amyloidosis compared to non-amyloidosis aetiologies.^44^ In another observational study, survival of patients were similar both in patients with FMF-amyloidosis and in other kidney transplant recipients.^45^ We could not find any reported case of renal transplantation due to PsA-related amyloidosis in the literature. Therefore, data for renal and patient’s outcome are insufficient in patients with kidney transplantation recipients due to PsA-related amyloidosis. In addition, the course of PsA and the role of immunomodulatory treatments in these patients is not known after kidney transplantation. In this report, both of the patients with kidneys transplantation had excellent graft function for more than five years of follow-up.

Our present study reveals the prevalence of amyloidosis in a large number of patients with PsA. Another strength of this study is in terms of establishing the clinical characteristics, long-term data and prognosis including renal transplantation experience of patients with PsA-related amyloidosis from a single centre. Additionally, we report the first case with successful treatment outcome by using a monoclonal antibody against IL-17A, secukinumab in a patient with refractory PsA and AA amyloidosis. The information of MEFV mutation status in patients with amyloidosis and its relationship with the clinical course was of importance. On the other hand, the retrospective design of the study and the absence of a control group were important limitations. The relatively low number of patients diagnosed with amyloidosis made it difficult to reach a definite conclusion.

## CONCLUSION

AA amyloidosis is a rare disorder complicating uncontrolled inflammatory diseases and the frequency of amyloidosis in patients with PsA is lower than FMF according to our previous results. On the other hand, it should be kept in mind that patients with PsA who have not received appropriate treatment for a long time and/or have refractory inflammatory response may develop AA amyloidosis. Polyarticular subtype and/or axial involvement of PsA may be risk factors in the development of PsA-related amyloidosis. The presence of accompanying factors such as heterozygosity for the *MEFV* variants and history of tuberculosis may facilitate the development of amyloidosis in patients with PsA. The *MEFV* gene variants may be screened in high-risk communities in patients with PsA-related AA amyloidosis. Favourable outcome was observed in patients with renal transplant recipients due to the PsA-related amyloidosis. Secukinumab may be a new treatment option for patients with PsA-related AA amyloidosis especially in patients who are refractory or intolerant to TNF inhibitors.
